# Population-dynamics focussed rapid rural mapping and characterisation of the peri-urban interface of Kampala, Uganda

**DOI:** 10.1016/j.landusepol.2009.12.003

**Published:** 2010-07

**Authors:** K. Makita, E.M. Fèvre, C. Waiswa, M.D.C. Bronsvoort, M.C. Eisler, S.C. Welburn

**Affiliations:** a Centre for Infectious Diseases, College of Medicine and Veterinary Medicine, Summerhall, University of Edinburgh, EH9 1QH Scotland, United Kingdom; b School of Biological Sciences and Centre for Infectious Diseases, University of Edinburgh, Ashworth Laboratories, Kings Buildings, EH9 3JT Scotland, United Kingdom; c Faculty of Veterinary Medicine, Makerere University, P.O. Box 7062, Kampala, Uganda; d Roslin Institute, University of Edinburgh, Easter Bush Veterinary Centre, EH25 9RG Scotland, United Kingdom

**Keywords:** Peri-urban, Agriculture, Kampala, Uganda

## Abstract

In developing countries, cities are rapidly expanding and urban and peri-urban agriculture (UPA) has an important role in feeding these growing urban populations; however such agriculture also carries public health risks such as zoonotic disease transmission. It is important to assess the role of UPA in food security and public health risks to make evidence-based decisions on policies. Describing and mapping the peri-urban interface (PUI) are the essential first steps for such an assessment. Kampala, the capital city of Uganda is a rapidly expanding city where the PUI has not previously been mapped or properly described. In this paper we provide a spatial representation of the entire PUI of Kampala economic zone and determine the socio-economic factors related with peri-urbanicity using a population-dynamics focussed rapid rural mapping. This fills a technical gap of rapid rural mapping and offers a simple and rapid methodology for describing the PUI which can be applied in any city in developing countries for wide range of studies.

## Introduction

In developing countries, cities are rapidly expanding; by 2025 it is estimated that over 50% of the population in those countries will reside in or around cities (FAO, 2002). Urban and peri-urban agriculture (UPA) has an important role in feeding these growing city populations (FAO, 2000); however it also carries public health risks such as transmission of zoonotic diseases (Flynn, 1999). It is thus important to study both the role of UPA in food security and the public health risks in order to seek the evidence-based best management policy of UPA.

As seen in the recovery from a food crisis in Cuba (Bourque, 2003), UPA can have a significant positive impact on food security. It also contributes to offer job opportunities (FAO, 2000) and its proximity to a city with high demand of food is a significant advantage for perishable food producers including livestock products such as milk (Brook et al., 2006). On the other hand, UPA has disadvantages as well: competition for resources especially access to land and water availability is intensive (Ellis and Sumberg, 1998). Crops can be contaminated with pathogens through irrigation and fertilization using untreated human and animal waste (Allen et al., 2006; Flynn, 1999) and with chemicals (Hardoy and Satterthwaite, 1997) and heavy metals (Bellows, 1999; Gertel et al., 2000; Huamain et al., 1999) through soils and irrigation waters. Zoonoses, defined as ‘diseases and infections that are naturally transmitted between vertebrate animals and man’ (WHO, 1959), can be transmitted to humans through consumption of livestock products, contact to wastes and occupational hazards such as working in farms, abattoirs and tanneries (Birley and Lock, 1999) and are growing concerns particularly in urban and peri-urban areas where dietary habits and animal feeding practice are changing (Steinfeld, 2004). As to livestock farming, however many risks it has, industrialised livestock production is expected to continue to play an important role in meeting the increasing demands for meat and milk (the ‘Livestock Revolution’) in the cities (Delgado et al., 1999; Devendra et al., 2005). On the other hand, livestock farming, even poultry farming which is the first step on the livestock ladder, is difficult to start for urban poor and the arena is known not to be appropriate for poverty-oriented development intervention due to constrains of limited profitability (Maxwell, 1995; Sumberg, 1998). In the long run, comparative advantage in production lies outside urban areas for the simple reason that land is cheaper (Ellis and Sumberg, 1998). Therefore two sets of policy support have been discussed; permitting urban poor the widest possible range of opportunities to piece together their livelihoods and enhancement of rural–urban interactions (Ellis and Sumberg, 1998) or in other words, urban–rural linkage (Allen and Julio, 2003).

The peri-urban interface (PUI) can be defined, in simple terms, as the areas around cities and towns characterised by rapid demographic, economic, environmental, social and cultural interactions and changes (Adam-Bradford et al., 2006). However, no single definition would fit all circumstances and situations (Simon et al., 2006) and there is no universally accepted definition (Birley and Lock, 1999) for the PUI. The rural, peri-urban and urban forms, in terms of system, uneven or lumpy, multidimensional continuum and it is far from a smooth, linear transition from urban to rural (Iaquinta and Drescher, 2001; Lynch, 2005). People communicate, exchange and travel across the urban–rural divide in order to maximize their livelihood opportunities (Lynch, 2005). Because of these reasons, little importance is now placed by researchers on attempting to measure the precise width of the PUI (McGregor et al., 2006) and general commonalities in peri-urban discourse emphasize the importance of transitional processes rather than the geographical location (Adam-Bradford et al., 2006; Binns, 1994; Brook and Dávila, 2000; Nunan, 2001; Rakodi, 1999; Simon et al., 2004; Tacoli, 2003).

The PUI suffers from pressures on resources, air and water pollution and land contamination, slum formation caused by migration (Douglas, 2006). Access to safe water is a basic human right (WHO, 2003), but national and international initiatives and commitments to improve access to water supply and sanitation in the developing world tend to neglect the peri-urban context (Allen et al., 2006), especially the peri-urban poor whose needs and practices often remain ‘invisible’ to the public sector (Hofmann, 2004). In the PUI, the institutional fragmentation, as a result of overlapping and changing local government institutions which are either still rural or have developed into urban but are inappropriate for peri-urban, is a unique problem (Allen, 2001; Lynch, 2005).

An adequate framework of the interventions for basic service provision in the PUI requires a better understanding of the impact of both spatial and non-spatial policies (Allen et al., 2006). Spatial policies include land-use changes, use and protection of renewable and non-renewable resources, pollution and waste management, spatial integration and environmental equity, and institutional reform with spatial dimension. Non-spatial policies include micro-economic, liberalization and transport policies and they are mainly of a sectoral nature, although they may indirectly exert an influence on the nature and flows of goods, people, services and waste between urban and rural areas (Dávila, 2006).

Although little attention is paid on geographical location of the PUI as explained above, understanding of the geography would contribute towards better understanding and interventions of the problems. A geographical information system (GIS) helps to facilitate the task of decision making as it tracks progress towards sustainability without using a single index of sustainability of the PUI (Diaz-Chavez, 2006). Also, the PUI is the area where large numbers of vector borne diseases such as malaria and water borne diseases are occurring due to certain productive activities and lack of water hygiene (Allen et al., 2006) and understanding geography is an essential tool for epidemiological research for such diseases to focus the targeted entry point of the intervention. The present study was indeed conducted as a starting point of an epidemiologic research for zoonoses in the PUI.

While vast literatures are available on the studies of the PUI, determination of geographical location of the PUI has been tackled by a limited number of studies in developing countries as well as developed countries. In Quebec, Canada, spatial and demographic dynamics in peri-urbanisation was studied using data between 1961 and 1991 (Paquette and Domon, 1999). In France, peri-urban territory was defined by analysing residential and agricultural land prices (Cavailhes and Wavresky, 2003). Another large scale research project called NEWRUR studied the interaction between urban expansion and surrounding rural areas in five European countries; France, Spain, UK, Germany and Greece between 2001 and 2004 (NEWRUR, 2004). All these studies were able to define the PUI because of rich and precise public sector data. However in developing countries such data may not be available.

The methodologies of determination of the PUI in developing countries have been well explored by the Natural Resources Systems Programme (NRSP) funded by the UK Department for International Development (DFID), in the studies of two cities: Kumasi in Ghana and Hubli-Dharwad in India (Adam, 2001; Brook and Dávila, 2000). The methods explored were satellite imagery (Landsat or SPOT) (Mather and Williams, 1996), colour infrared aerial digital photographic (ADP) system survey, the micro-light platform—a low cost and compact aircraft, the balloons platform (D'Souza and D'Souza, 2000; Brook and Dávila, 2000), high resolution videography (HRV) (Curr and Curr, 1996) and integration of rapid rural assessment (RRA)/participatory rural appraisal (PRA) and geographic information systems (GIS) called rapid rural mapping (Brook and Dávila, 2000; D'Souza and D'Souza, 2000). Several studies on urban sleeping sickness also used satellite imagery (SPOT 4) to delineate urban, peri-urban and rural areas in Kinshasa, Democratic Republic of Congo (Deken et al., 2005; Robays et al., 2004; Simo et al., 2006). The PUI is difficult to determine due to the complex and heterogeneous landscape (Adam, 2001; Drechsel et al., 1999) but the rapid rural mapping used in Kumasi, Ghana and in Hubli-Dharwad, India have overcome this problem by classifying the level of urbanicity of randomly selected villages. Iaquinta and Drescher (2000) described the dynamic, interactive and transformative processes which take place in the PUI by classifying it into 5 types: village PU (rural places with urban consciousness), diffuse PU (in-migration from several places), chain PU (reconstituted, in-migration from a single place), in-place PU (traditional, in situ urbanisation) and absorbed PU (traditionalism with succession/displacement). As to the dynamics of the flow of migration, rural to urban migration is dominant among a variety of movements between rural and urban areas (Lynch, 2005).

Kampala, the capital city of Uganda, is a rapidly expanding city which comprises more than 39% of Uganda's urban population (Nkurunziza, 2008) and there are a number of studies relating to UPA in this setting (King’ori, 2004; Maxwell, 1995) and many unpublished pieces of works (Kimeze, 2005); however the PUI has not mapped yet. The present study attempts to determine the PUI of Kampala using a population-dynamics focussed rapid rural mapping and to discuss the standardization of the method applicable to other PUIs.

## Materials and methods

### Study sites

**Fig. 1:**
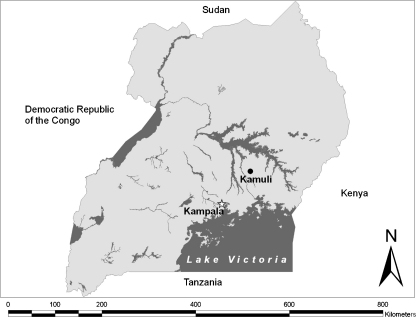
Map of Uganda showing locations of Kampala and Kamuli.

Two study sites, Kampala (latitude 0.31573 north and longitude 32.57726 east), the capital city of Uganda, and Kamuli (latitude 0.94511 north, longitude 33.12374 east and approximately 100 km northeast of Kampala) were selected for this study (Fig. 1). Kamuli economic zone, a small town in a rural area which is too far to be directly influenced by urbanisation of the capital city, was selected as the control to the Kampala economic zone to test the hypothesis that urbanisation is observed in any size of towns/cities.

### Sampling methods

Stratified random sampling was used to determine the sampling sites of this study. Strata were LC3s (Local Council 3: Sub-Counties), and sampling units were LC1s (Villages). Uganda has an administrative system consisted with 5 layers: District (LC5), County (LC4), Sub-County (LC3), Parish (LC2) and zone/village (LC1) (United Nations, 2004). *LC3s selection*: in the Kampala economic zone, LC3s where more than 50% of the LC1s were located in an area between 5 and 20 km radii from the city centroid were selected. In the Kamuli economic zone, LC3s were selected with the same manner from the areas within 10 km radius circle from Kamuli Town centre but excluding Kamuli Town Council LC3. *LC1s selection*: to make sample LC1s represent the selected LC3s well, they were randomly selected. The sample size was calculated using Epi info version 3.3.2. In the Kampala economic zone, the expected frequency was set as 50% so that necessary sample size would be largest, because the proportion of LC1s categorised as peri-urban could not be estimated *a priori*. Absolute precision and confidence interval were set as ±10 and 95%, respectively. In the Kamuli economic zone, expected frequency was set as 90%, as most of LC1s were thought to have rural feature. Absolute precision and confidence interval were set as ±10 and 95.0%, respectively. After the determination of the sample size, proportional allocation was used to determine the sample size of LC1s in each LC3 and the sample LC1s were randomly selected. In the Kampala economic zone, 87 of 790 LC1s in 10 LC3s and in the Kamuli economic zone, 30 of 220 LC1s in 3 LC3s were selected.

### Village Characterisation Survey (VCS)

**Fig. 2:**
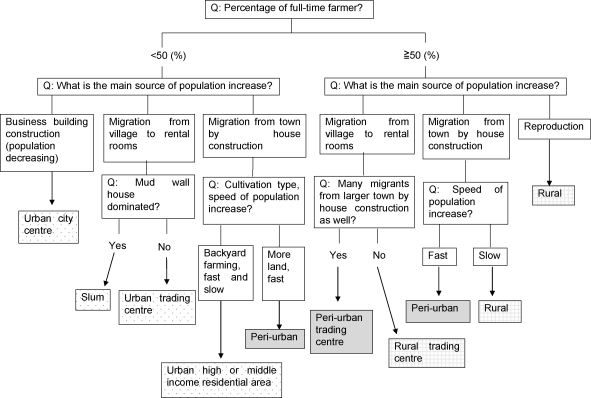
Decision tree model for urbanicity and development type classification of LC1s.

The Village Characterisation Survey (VCS) is a survey to classify the LC1s into urban, peri-urban and rural groups by conducting rapid rural appraisals (RRA) with key-informants and to determine their socio-economic characteristics by following administration of structured interviews using a questionnaire on the same day. In addition to the RRA and interviews, observed aspects, for example, features of dominant buildings and vegetation were recorded. The LC1 locations were recorded with a hand-held GPS (Garmin, Olathe, KS, USA). All GPS readings were taken at the LC1 office or the village leader's residence where interviews were performed. This VCS is a rapid rural mapping described in the study in Kumasi (Adam, 2001) but the details as to how the level of urbanicity was determined were not given in the literature. The definitions of the level of urbanicity and development types were therefore determined in the present study (Table 1) from the findings of the RRA. The common findings were the people's flow in urbanisation which starts from rural areas as migration to small and cheap rental rooms in trading centres and slums in urban areas. When people successfully find a job to earn enough wages, they move to better rented rooms in urban residential areas or peri-urban areas by purchasing a plot to construct a house. Rich urban residents also move to peri-urban areas by purchasing more land and constructing bigger houses; this is how the city grows in Kampala. Urbanisation is a dynamic process. At first, rural areas are static in terms of population change. When the rural area changes to a peri-urban area, house construction and the migration from the city start and the speed of the population change soon becomes high. The speed will be slow again when most of the agriculture fields are replaced with houses; transformed into urban residential areas. The population does not increase in trading centres when the population density becomes very high. Such trading centres are still receiving immigrants from rural but at the same time, ‘sending’ people out to residential and peri-urban areas. These findings were used to develop a decision tree model of urbanicity classification of LC1s (Fig. 2). The classification process starts from calculation of the percentage of full-time farming households and then in-migration and its direction is asked. Observational and agricultural information were also used to assist the classification process qualitatively. The questionnaire used in the VCS was developed selecting the indicators of peri-urban settlements found by Adam (2001) and Delgado et al. (1999) in West Africa (Table 2).

The VCS, the combination of RRA and structured interviews, was conducted from 23rd September to 8th November 2005 both in the Kampala and the Kamuli economic zones. The decision tree model was developed during the survey and the level of urbanicity was judged with the model using the recorded information at the RRA. As far as possible, the LC1 leaders and other committee members were interviewed together. If the LC1 leader was not available, and other committee members could not answer the questions, the LC1 leader was contacted later by mobile phone or direct revisit.

Also, land prices of sample LC1s in the Kampala economic zone in 2004 were investigated from the land transaction records at the Valuation Division of the Ministry of Water, Land and Environment, Uganda.

### Statistical analysis

To find the socio-economic factors related with urbanicity, the results of interviews only of the Kampala economic zone were compared among urban, peri-urban and rural LC1s. When a significant difference was found among three urbanicity groups, which means at least one group is significantly different, each two adjacent groups (urban and peri-urban LC1s, and peri-urban and rural LC1s) were compared. Continuous data (number of households per square kilometre, Euclidean distance from city centroid, transportation cost to Kampala Taxi Park and land price) were tested using One-Way ANOVA. To calculate the number of households per square kilometre, LC2 area data were obtained from Land and Survey Office, Uganda and LC1 areas were calculated by dividing the LC2 (Parish) area by the number of LC1s consisting the LC2. Data were transformed with the transformation parameter *λ* (lambda) obtained by Box–Cox transformations (Box and Cox, 1964; Crawley, 2002) before performing One-Way ANOVA when the errors were not normally distributed. The time to nearest trading centre was analysed using a Generalised Linear Model (GLM) with quasipoisson errors and obtained results were back-transformed using exponential (Crawley, 2002). The perception of pollution in rank (0: none, 1: feel pollution, 2: feel very much) was tested using Kruskal–Wallis Test in MINITAB 14.1. The percentage of full-time farming households, the percentage of LC1s having land disputes, schools and public facilities were tested using Chi-square test. A 95% confidence interval was calculated for all of the percentages using Chi-square test with one proportion.

The relationships between distance from city centroid and the proportion of LC1s with public facilities were analysed using a GLM with binomial errors. Fitted prediction and 95% confidence interval lines of the relationship between proportion of LC1s having the facilities and the distance from city centroid were obtained using the model. The statistic software R, version 2.4.1, was used for all tests other than the Kruskal–Wallis Test.

## Results

### Classification of LC1s into urban, peri-urban and rural groups in the Kampala and Kamuli economic zones

In the Kampala economic zone, 59 of 87 LC1s were classified into urban (67.8%), 11 were into peri-urban (12.6%), and 17 were into rural (19.5%) by the VCS. Middle income residential areas were the most predominant development type and accounted for 37.3% (22 LC1s) of urban LC1s, and the second predominant type was trading centre (17 LC1s, 28.8%). One of 17 rural LC1s classified into university/institute was a prison. Twelve LC1s (11 urban LC1s and 1 rural LC1) were unable to be interviewed as there were no residents (university/institution), or houses were fenced and guarded in very high income residential areas. All adults were absent in a compound for doctors and nurses of a hospital in a middle income residential area LC1 and only speed of population change and direction of migration were asked to teen-agers. Therefore, socio-economic characteristics were analysed for only 74 LC1s (47 urban, 11 peri-urban and 16 rural LC1s). In the Kamuli economic zone, out of 30 LC1s, 2 LC1s were peri-urban (6.7%, 95%CI: 1.2–23.5) and 28 were rural (93.3%, 95%CI: 76.5–98.8) and there was no urban LC1. One of 2 peri-urban LC1s (50%) and 5 of 28 rural LC1s (17.9%) were trading centres.

### Spatial distribution of urban, peri-urban and rural LC1s

**Fig. 3:**
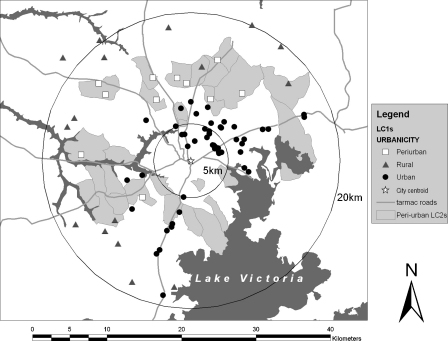
Map of urban, peri-urban and rural LC1s in Kampala economic zone and the spatial distribution of the peri-urban interface (PUI). Highlighted areas, the PUI, include LC2s classified as peri-urban during the additional studies.

Fig. 3 shows the spatial distribution of classified urban, peri-urban and rural LC1s in the Kampala economic zone and the spatial distribution of the PUI. Since the PUI is vague and dynamic areas in reality, it is very difficult to show exact areas; therefore in this figure the LC2s including peri-urban LC1s or LC2s judged to have peri-urban feature were highlighted (gray areas) as the PUI. In Kampala, city centroid was surrounded by urban LC1s and as the distance from the city centroid increased, the level of urbanicity decreased to peri-urban and rural. Euclidean distance (km) from city centroid to LC1s was significantly shorter in urban LC1s (6.4, 95%CI: 4.7–8.7) than peri-urban LC1s (12.1, 95%CI: 10.5–13.9; *F* = 25.34, df = 1, error = 68, *p* < 0.001) and longer in rural LC1s (17.0, 95%CI: 14.6–19.7; *F* = 19.55, df = 1, error = 29, *p* < 0.001) than peri-urban LC1s (Table 3). The PUI highlighted in Fig. 3 includes peri-urban LC2s classified during additional studies (Makita et al., 2008; and the other studies in preparation). The PUI surrounded the urban areas in a concentric fashion but the distance between city centroid and the PUI was shorter in the southern parts close to Lake Victoria than the other parts.

In the Kamuli economic zone, 2 peri-urban LC1s were located at 0.8 and 1.7 km from the town centre (mean 1.3 km), and the other rural LC1s surrounded them.

### Socio-economical characteristics of urban, peri-urban and rural areas

Hereafter socio-economical characteristics of the PUI are described only for the Kampala economic zone because it contained LC1s with all levels of urbanicity. The PUI in the Kampala economic zone was characterised by the middle range of several variables: household density (number of households per square kilometre), Euclidean distance from city centroid, transportation cost to Kampala Taxi Park (Table 3) and percentage of full-time farmers. The percentage of full-time farmers was used in the decision tree model, but it was just to separate first tree either it is more than and equal to 50% or less than 50%, and either branch can lead to peri-urban. Therefore doing statistics on the percentage of full-time farmers is not a circular argument. It took longer time to go to the nearest trading centre on foot from rural LC1s than from peri-urban LC1s, but the time was not significantly different between urban and peri-urban LC1s. Pollution was perceived in urban and peri-urban LC1s but not in rural LC1s and even in urban and peri-urban LC1s, the perception was not strong. Land price in urban LC1s was significantly higher than in peri-urban LC1s, but the price in peri-urban LC1s was not significantly different from rural LC1s.

Table 4 shows the binomial data. Although peri-urban LC1s marked the largest proportions, there was no significant statistical difference among three urbanicity groups in the proportion of LC1s with land disputes between old residence and new comer or land disputes between agriculture and non-agriculture land use. Also no significant difference was found in the proportions of LC1s having a public or private primary school or private secondary school. Public secondary schools were seen only in urban LC1s. The proportion of LC1s having road light was not significantly different between urban and peri-urban LC1s and there was no road light in rural LC1s. Piped water was more commonly supplied in urban LC1s (91.5%) than in peri-urban LC1s (54.5%) and less supplied in rural LC1s (6.3%). The provision of sewage pipe and garbage collection service was seen only in urban LC1s. Electricity was supplied in all urban LC1s and in peri-urban areas (81.8%), it was more commonly supplied than in rural areas (25.0%). Recent improvement of public facilities was more frequent in peri-urban areas (72.7%) than in rural areas (18.8%) but was not significantly different from urban areas (46.8%). The most common facility improved was piped water supply (22/33 LC1s, 66.7%).

### The relationships between the distance from city centroid and the proportions of LC1s having public facilities

**Fig. 4:**
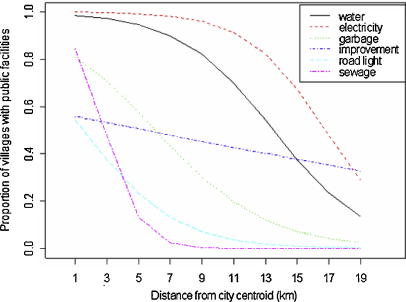
Relationship between the distance from city centroid and proportion of LC1s with public facilities. The proportions of LC1s having piped water supplied and electricity declined sharply around the peri-urban interface (12.1 km, 95%CI: 10.5–13.9), especially piped water supply.

Fig. 4 shows the relationships between the Euclidean distance from city centroid and the proportions of LC1s having public facilities. The factors were piped water supply, electricity, garbage collection service, recent improvement of public facilities, road light and sewage pipe. All lines shown are fitted lines by GLM with binomial errors. The proportions of LC1s having piped water supply (slope = −3.1, *p* = 0.01), electricity (slope = −5.8, *p* < 0.001), garbage (slope = −2.6, *p* = 0.03), road light (slope = −3.2, *p* = 0.01) and sewage pipe (slope = −5.0, *p* = 0.001) declined significantly with the increase of the distance from city centroid. The proportion of LC1s with recent improvement of public facilities showed a weaker relationship with the increase of the distance (slope = −2.0, *p* = 0.08). Among these public facilities related with the distance from city centroid, piped water supply and electricity declined sharply around the peri-urban interface (12.1 km, 95%CI: 10.5–13.9, Table 3), especially piped water supply.

**Fig. 5:**
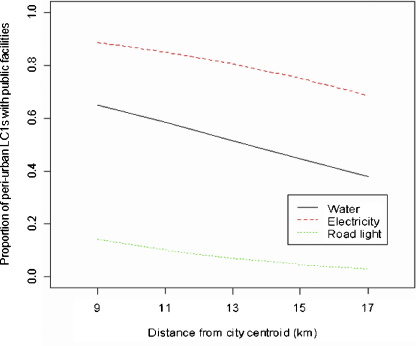
Relationship between the distance from city centroid and proportion of peri-urban LC1s with public facilities. The proportions of LC1s with any of piped water supply (slope = −0.5, *p* = 0.65), electricity (slope = −0.4, *p* = 0.71), and road light (slope = −0.5, *p* = 0.68) did not decline significantly with increase of the distance from city centroid.

However, when only peri-urban LC1s were analysed, the proportions of LC1s with any of piped water supply (slope = −0.5, *p* = 0.65), electricity (slope = −0.4, *p* = 0.71), and road light (slope = −0.5, *p* = 0.68) did not decline significantly with increase of the distance from city centroid (Fig. 5). There was no garbage collection service or sewage pipe in the peri-urban LC1s.

## Discussion

The present study used rapid population change due to migration from the city by house construction as the indicator of peri-urbanicity in the decision tree model developed and it is actually the dynamic part of the PUI defined by Iaquinta and Drescher (2000). The key-informants and participants of RRA depicted the flow of people in urbanisation—the real life of urban migration well. Most of the slums in Kampala were located in a valley or a swamp which were easily flooded with rains but the poor lived in overcrowded conditions in such slums because the rooms were the only affordable option. Not all people may escape from the poor livelihoods, but people who successfully find a job or started a business gradually move to a better room and finally buy land and construct a house in peri-urban areas. Compared with Kampala, the size of Kamuli Town was very small, but urbanisation and expansion of the small town were observed; migration from rural to urban was mentioned and new houses were being constructed in two peri-urban LC1s. These findings in the present study may be universal and the decision tree model of urbanicity classification may be applicable to any size of village, town and cities in any developing country. It should be useful to modify the present model or methodology reflecting the type of peri-urbanisation described by Iaquinta and Drescher (2000) for the application to the other PUIs.

In the field, the interface between middle income residential areas and peri-urban areas was always difficult to judge even with the visual information. This is quite natural because the wave of peri-urbanisation always moves from the city outwards with a continuous vague gradation. Researchers should bear in mind that the observation on the ground may give a biased impression. Agricultural fields can be hidden behind crowded settlements as peri-urban areas are heterogeneous. Aerial photography data can be an alternative or complementary tool to direct observation. However, direct visits and interviews regarding population change were still thought to be very important to determine the PUI unless fine demographic data is available.

**Fig. 6:**
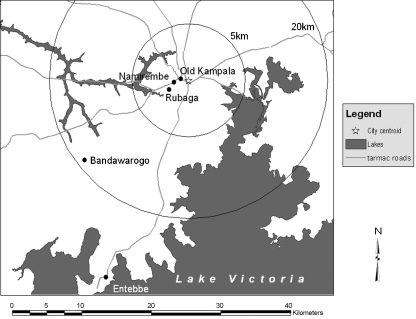
Map showing locations of palaces of Buganda Kingdom and Kampala Hill (Old Kampala). Mengo Hill is not indicated but it is located in the east of Namirembe Hill and slightly west of the Entebbe Road (gray line towards Entebbe).

Spatial distributions of urban, peri-urban and rural LC1s in Kampala and Kamuli enabled us to understand the size and the shape of the city and the town. Initially the urban areas of Kampala were thought to be extended towards Entebbe, located at 35 km south-southwest from Kampala (Fig. 6), which was the capital city under British colonial rule from 1893 to 1968 (Southall and Gutkind, 1957). However, the urban areas of Kampala were formed in a concentric fashion and the distance between the PUI and the city centroid was even shorter towards Entebbe than the other directions. This might be because there is Lake Victoria in the south of Kampala, at which stops Entebbe Road at Entebbe, and the areas might not be attractive for the development. The history of the urban formation of Kampala helps to understand the shape of the PUI (Fig. 6). The history of Kampala started sometime between 1862 and 1875. When Speke who discovered the source of the Nile River visited the palace in 1862, the royal palace of the Buganda Kingdom, the largest kingdom of current Uganda, was located in Bandawarogo (Speke, 1863), 20 km southwest of current Kampala city centre, and the Christian missionary, Stanley found the palace in Rubaga, a part of current Kampala City in 1875 (Southall and Gutkind, 1957). In 1890, Lugard, sent by the Imperial British East Africa Company, found the palace in Mengo Hill of current Kampala (Perham, 1956) and he soon seized the Kampala Hill (Colvile, 1895; Rowe, 1969); this was a beginning of current Kampala City. The capital of Uganda was built on four hills, Mengo, Rubaga, Namirembe and Kampala, the first three being occupied by the king, the Catholic and Protestant missions, respectively, while the last was selected by the officers of the Imperial British East Africa Company as the site for their fort (Colvile, 1895; Southall and Gutkind, 1957). The development of Kampala and Entebbe started almost same time but Kampala remained as the economical centre although Entebbe was the administrative centre; Kampala expanded in a concentric fashion without much influence of Entebbe (Southall and Gutkind, 1957).

From the analyses of socio-economic factors related to the PUI of the Kampala economic zone, peri-urban LC1s were characterised by the middle range of several variables: household density, Euclidean distance from city centroid, transportation cost to Kampala Taxi Park and percentage of full-time farming household. The finding on intermediate range of transportation was consistent with peri-urban areas in Kumasi, Ghana (Adam, 2001).

The walking time to the nearest trading centre revealed that life in the peri-urban interface was as convenient as urban areas in terms of the access to commodities and the time took significantly longer in rural areas. Pollution was perceived both in urban and peri-urban LC1s to a similar degree but not perceived in rural in the Kampala economic zone. The most popular complaint was plastic bags thrown away. In Hubli-Dharwad, India, peri-urban villagers feared increased air and water pollution from the growing city and loss of common land and open space (Patil, 1999), and peri-urban interface was the chosen location for the cities landfills, where waste pickers operate (Brook and Dávila, 2000). In the present study in Kampala also, a peri-urban LC1 leader complained about nearby urban waste landfill; however, statistics did not show strong perception of pollution among people in peri-urban Kampala. In Kumasi, Ghana, environmental degradation was less recognised in peri-urban settlements than elsewhere as a recent negative change (Adam, 2001). Perception of pollution may not be a particular indicator of peri-urbanicity.

Land price was tested for the similarity with the description by Cavailhes & Wavresky (2003) that farmland prices fall sharply close to the city and then gently further away in France. In Kampala, the results showed a similar finding that the mean land price in urban areas was significantly higher than peri-urban areas and rural land price did not differ from that of peri-urban areas. Land price may be useful to know the border between urban areas and the PUI.

The proportions of LC1s having piped water supply, and provision of electricity sharply declined over the distance from city centre around the PUI, and in peri-urban LC1s, these proportions did not change over distance from city centroid. It was interpreted that the PUI is where piped water supply and electricity are dynamically being provided. As the proportion of peri-urban LC1s with electricity was large, electricity may be provided just before peri-urbanisation starts. Piped water was supplied to around half of the peri-urban LC1s and it was the most common facility recently improved overall; it may be the best indicator of the PUI of Kampala. Interestingly, in Kumasi, Ghana, peri-urban settlement had recent improvements in health, electricity and public toilet facilities (Adam, 2001). As he mentioned, electricity should be provided a few years before the wave of peri-urbanisation reaches. In the PUI of Hubli-Dharwad, India, electricity is extended to the more accessible villages, where provision is between 80 and 100%, and piped water is extended to some peri-urban villages (Brook and Dávila, 2000; Hunshal and Nidagundi, 1997). These findings can be similar in other cities in developing countries. There were some other sociological factors which were not consistent with the studies carried out by the other studies. For example in Kumasi, peri-urban settlements had more land disputes and junior secondary schools than the other settlements (Adam, 2001), but in Kampala, although the proportions of LC1s with these factors were the highest in peri-urban areas, they statistically did not. As to the small numbers of land disputes, the peri-urban respondents explained that they were due to tenure of *bibanja*, which we need to refer the history of land ownership. In pre-colonial Buganda, most land was nominally controlled by the Kabaka, the King. In 1900, the British signed an agreement in which they gave 8958 square miles to the Kabaka, the royal family and several thousand top Buganda chiefs as freehold – known as *mailo* land (from the word ‘mile’) – and allocated the rest, 9000 square miles of ‘waste and uncultivated land’ to the Protectorate as Crown land (Green, 2005). *Mailo* land was legally inheritable (Richards, 1963) and due to its privileges such as freedom from tax and demand for titled land, the market of *Mailo* land was emerging (Porter, 2001). There was another issue that landowners increased land rents (*busuulu*) and commodity rents (*envujjo*) to unreasonable level. To stabilize these situations, *Busuulu* and *Envujjo* Law was passed in 1928; this law secured the rights of tenancy which is inheritable and regulated annual land and commodity rents. The plots of these lands are called as *bibanja* (single form is *kibanja*) (Green, 2005; Porter, 2001). Although these private lands were taken by Milton Obote in 1966 and turned into leasehold property by Idi Amin in 1975, landlords continued to maintain their place in Bugandan society by leasing land to poorer tenants in an informal manner (Green, 2005; Karlström, 1999). The Land Act, introduced by Yoweri Museveni in 1998, formalized the rights of *bibanja* again but it created much controversy in Buganda (Porter, 2001). The detailed discussion on this Act is not the scope of the present paper but because of this Act, rights of *bibanja* became legal and it was contributing to the stability of land tenure in the study sites.

The present study enabled to map the PUI of Kampala by the combination of understanding population dynamics and use of GIS. The rapid rural mapping of villages had been introduced (Brook and Dávila, 2000; D'Souza and D'Souza, 2000) but the explicit methodology which classifies villages into urban, peri-urban and rural has not been available, and the present study filled this gap. Although there is a view that geographical location of the PUI is not important, the methodology developed in the present study would contribute to wide range of peri-urban researches as well as decision makers for strategic planning of the PUI, especially in the resource limited countries.

## Figures and Tables

**Table 1:** Definitions of levels of urbanicity and development types of LC1s.

1. Levels of urbanicity
*Urban*:
Densely populated areas and the main agricultural activity is backyard farming in small plots.
*Peri*-*urban*:
Transition areas from rural to urban, the speed of population increase is high, migration is from city or town by house construction, and there is still space for crop cultivation.
*Rural*:
Static areas before urbanisation starts, people are mostly dependent on agriculture, speed of population increase is slow and the main source of the increase is reproduction.

2. Development types
*City centre*: Dominated by business buildings.
*High income residential area*: Large luxurious houses with high fence.
*Slum area*: Crowded by low income residents, characterised by mud wall houses.
*Middle income residential area*: Residential area between above two types.
*Trading centre*: Trading market with shops and restaurants along a road.
*University*/*institution*: whole area of the LC1 is occupied with a university, or an institution.
*Peri*-*urban*: Rapid population increase due to the migration from the city or town by house construction.
*Rural*: The population change of peri-urbanisation has not started yet.

**Table 2:** Contents of the interviews with the village leaders.

1. Sociological information
a. Total number of households, number of full-time farming households
b. Cost of transportation to Kampala city centre by public means
c. Time to nearest trading centre on foot
d. Provision of public facilities (electricity, piped water supply, sewage pipe, garbage collection service, road light)
e. Recent improvement of public facilities (less than 5 years)
f. The number of ongoing land disputes between old residents and new comer, agriculture and non-agriculture
g. The number of schools (public and private primary school and secondary school)
h. Perception of pollution (no, feel, very much)
i. Speed of population change
j. Direction of migration (from village or city)

2. Agricultural information
a. Numbers of tomato, cooking banana, maize, rice, green vegetable farmers
b. Numbers of those farmers who sell to markets in Kampala
c. Numbers of those farmers who sell to nearby trading centre
d. Destination of the products
e. Purpose of their farming
f. Numbers of large scale crop and vegetable farmers with more than 10 acre land

**Table 3:** Socio-economic factors related to urbanicity (continuous and ranked data).

Factor	Mean with 95%CI			Test statistics	*p*-value
	Urban	Peri-urban	Rural		
Number of households/km^2^ (log)	3.0 ± 1.0	2.2 ± 0.7	1.8 ± 0.6	UP: *F*_1,57_ = 21.37	*p* < 0.001
Back-transformed: number/km^2^	1047 (96–11, 481)	174 (39–776)	62 (16–240)	PR: *F*_1,25_ = 13.56	*p* = 0.001

Distance from city centroid (m, sqrt)	80.0 ± 11.3	110.2 ± 7.9	130.5 ± 9.7	UP: *F*_1,68_ = 25.34	*p* < 0.001
Back-transformed: km	6.4 (4.7–8.3)	12.1 (10.5–13.9)	17.0 (14.6–19.7)	PR: *F*_1,26_ = 19.55	*p* < 0.001

Transportation cost to Kampala (log)	6.3 ± 0.2	6.7 ± 0.2	7.2 ± 0.3	UP: *F*_1,56_ = 13.29	*p* < 0.001
Back-transformed: Uganda Shillings	545 (446–665)	812 (665–992)	1339 (992–1808)	PR: *F*_1,24_ = 15.93	*p* < 0.001

Time to nearest trading centre (log)	2.2 ± 1.2	1.8 ± 1.2	3.4 ± 1.3	UP:	*p* = 0.488
Back-transformed: min	9.2 (2.8–30.2)	6.1 (1.8–20.7)	28.7 (8.2–100.6)	PR:	*p* = 0.010

Perception of pollution (rank: 0–2)^a^	1.0	1.0	0.0	H_2_ = 15.56	*p* < 0.001
Land price (0.2nd power)	61.3 ± 5.6	49.1 ± 5.5	44.2 ± 7.3	UP: *F*_1,51_ = 19.66	*p* < 0.001
Back-transformed: million Shil/acre	866 (536–1340)	285 (158–485)	169 (68–362)	PR: *F*_1,22_ = 2.11	*p* = 0.16

UP: between urban and peri-urban, PR: between peri-urban and rural, sqrt: square root.

a Values for perception of pollution are median.

**Table 4:** Socio-economic factors related to the level of urbanicity (binomial data).

Factors	Percentage with 95%CI (positive response)			Test statistics
	Urban (*n* = 47)	Peri-urban (*n* = 11)	Rural (*n* = 16)	
Land disputes between old resident and new comers	23.4: 12.8–38.3 (11)	36.4: 12.4–68.4 (4)	12.5: 2.2–39.6 (2)	*x*^2^ = 2.1, df = 2, *p* = 0.35
Land disputes between agricultural and non-agricultural land use	14.9: 6.9–28.9 (7)	45.5: 18.1–75.4 (5)	12.5: 2.2–39.6 (2)	*x*^2^ = 6.0, df = 2, *p* = 0.05
Public primary school	36.2: 23.1–51.5 (17)	63.6: 31.6–87.6 (7)	50.0: 28.0–72.0 (8)	*x*^2^ = 3.1, df = 2, *p* = 0.21
Private primary school	55.3: 40.2–69.5 (26)	72.7: 39.3–92.7 (8)	43.8: 20.8–69.4 (7)	*x*^2^ = 2.2, df = 2, *p* = 0.33
Public secondary school	6.4: 1.7–18.6 (3)	0.0: 0.0–32.1 (0)	0.0: 0.0–24.1 (0)	NA
Private secondary school	31.9: 19.5–47.3 (15)	54.5: 24.6–81.9 (6)	12.5: 2.2–39.6 (2)	*x*^2^ = 5.4, df = 2, *p* = 0.07
Road light	17.0: 8.1–31.3 (8)	9.1: 0.5–42.9 (1)	0.0: 0.0–24.1 (0)	UP: *x*^2^ = 0.04, df = 1, *p* = 0.85

Piped water supply	91.5: 78.7–97.2 (43)	54.5: 24.6–81.9 (6)	6.3: 0.3–32.3 (1)	UP: *x*^2^ = 6.9, df = 1, *p* = 0.01
				PR: *x*^2^ = 5.6, df = 1, *p* = 0.02

Sewage pipe	14.9: 6.9–28.9 (7)	0.0: 0.0–32.1 (0)	0.0: 0.0–24.1 (0)	NA
Garbage collection	51.1: 36.3–65.7 (24)	0.0: 0.0–32.1 (0)	0.0: 0.0–24.1 (0)	NA
Provision of electricity	100: 90.6–100 (47)	81.8: 47.8–96.8 (9)	25.0: 8.3–52.6 (4)	PR: *x*^2^ = 8.4, df = 1, *p* = 0.004

Recent improvement of public facilities	46.8: 32.4–61.8 (22)	72.7: 39.3–92.7 (8)	18.8: 5.0–46.3 (3)	UP: *x*^2^ = 1.5, df = 1, *p* = 0.2
				PR: *x*^2^ = 5.8, df = 1, *p* = 0.02

UP: comparison between urban and peri-urban; PR: comparison between peri-urban and rural.
